# A prospective and randomized clinical trial evaluating the effectiveness of ART restorations with high-viscosity glass-ionomer cement versus conventional restorations with resin composite in Class II cavities of permanent teeth: two-year follow-up

**DOI:** 10.1590/1678-7757-2020-0609

**Published:** 2021-03-01

**Authors:** Rafael MENEZES-SILVA, Sofia R Maito VELASCO, Eduardo BRESCIANi, Roosevelt da Silva BASTOS, Maria Fidela de Lima NAVARRO

**Affiliations:** 1 Universidade de São Paulo Faculdade de Odontologia de Bauru Departamento de Materiais Dentários, Endodontia e Dentística BauruSP Brasil Universidade de São Paulo, Faculdade de Odontologia de Bauru, Departamento de Materiais Dentários, Endodontia e Dentística, Bauru, SP, Brasil.; 2 Universidade de São Paulo Faculdade de Saúde Pública São PauloSP Brasil Universidade de São Paulo, Faculdade de Saúde Pública, São Paulo, SP, Brasil.; 3 Universidade Estadual Paulista Instituto de Ciências e Tecnologia São José dos CamposSP Brasil Universidade Estadual Paulista (UNESP), Instituto de Ciências e Tecnologia, São José dos Campos, SP, Brasil.

**Keywords:** Permanent dentition, Atraumatic restorative treatment, Glass-ionomer cement, Resin composite, Clinical trial

## Abstract

**Objective:**

To compare the effectiveness of ART restorations using High Viscosity Glass-ionomer cement (HVGIC) with conventional restorations using resin composite in Class II cavities of permanent teeth, in a 2-year follow-up.

**Methodology:**

Seventy-seven restorations were made with each restorative material, Equia Fil-GC Corporation (ART restorations) and Z350-3M (conventional restoration), in 54 participants in this parallel and randomized clinical trial. Restorations were evaluated at 6 months, 1 and 2 years using the ART and the modified United States Public Health Service (USPHS) criteria. Chi-square test and Survival Analysis (p<0.05) were used for statistical analysis.

**Results:**

The success rates for ART restorations were 98.7% (6 months) and 95.8% (1 year) for both criteria. At 2 years, success rate was 92% and 90.3% when scored by the modified USPHS and ART criteria (p=0.466), respectively. The success rates for conventional restorations were 100% (6 months), 98.7% (1 year) and 91.5% (2 years) for both assessment criteria. ART restorations presented a lower survival rate by the criterion of ART (83.7%) when compared to the modified USPHS criterion of (87.8%), after 2 years (p=0.051). The survival of conventional restorations was 90.7% for both evaluation criteria.

**Conclusion:**

At the 2-years follow-up evaluation, no statistically significant difference was observed between the success rate of ART restorations with HVGIC compared to conventional restorations with resin composite in Class II cavities of permanent teeth.

## Introduction

Currently, the main alternatives of direct restorative material to substitute dental amalgam are resin composite and polyalcenoate-based materials, with the glass-ionomer cements (GIC) being the most biomimetic ones.^1^ In contrast to resin composite, the GIC presents a coefficient of linear thermal expansion similar to tooth structures and it releases fluoride, characterizing its anticariogenic property.^2^

GICs emerged as the most suitable restorative materials in early studies on the impact of ART to oral health.^3^ Today, ART is widely accepted by the international scientific community and used worldwide.^4^

Although High Viscosity Glass-ionomer cement (HVGIC) is the material of choice for ART restorations, there is still room for improvements. Thus, some authors have proposed and tested additional retention in cavities restored with GICs to provide greater longevity to restorations in permanent teeth.^5,6^ Further, encapsulating HVGICs led to *in vitro* increased flexural strength,^7^ with possible positive influences to the restorative treatment.

According to a systematic review,^8^ it cannot be suggested that resin composite has higher failure rates and risk for secondary caries than amalgam restorations due to the weak scientific evidence. Thus, with the Minamata Convention and the reduction in the use of mercury in several fields, including dentistry, resin composite restorations are considered viable alternatives to amalgam restorations.^9^ Therefore, in studies looking for new restorative alternatives, resin composites must be considered control.

Mickenautsch^10^(2016) investigated the state of the art of direct restorations in posterior permanent teeth applying HVGICs. The author concluded that ART restorations showed similar clinical performance to amalgam restorations. Kielbassa, et al.^9^ (2017) proposed that the promising HVGIC Equia Fil could be an option to dental amalgam in countries where health services do not cover resin composites or in cases of allergy to polymers.

Considering the lack of randomized and parallel clinical trials with high internal validity comparing resin composites and HVGICs, both in deciduous teeth and permanent teeth, it is difficult to indicate the superiority of a material,^11^ especially considering the substitution for dental amalgam. Few studies have investigated the clinical performance of multiple-surface restorations using GICs and resin composites in permanent teeth.^12-14^ Evaluating restorations performed with HVGICs under the ART approach would provide important data considering three aspects: testing a substitute for dental amalgam, the ART approach being a more socially available technique due to the non-use of electrical equipment, and ART might be an important approach in COVID era as it does not generate aerosols.^1,8,15-17^

Therefore, the objective of the present study was to compare ART restorations with HVGIC versus conventional restorations with resin composite in Class II cavities of permanent teeth over a period of 2 years. Previous follow-up data have been published elsewhere.^18^ That study presented similar success rates considering both approaches, granting longer evaluation assessments.

The tested null hypothesis assumes there is no difference after 2 years on the effectiveness of ART restorations with HVGIC compared to conventional restorations with resin composite.

## Methodology

This is a prospective and randomized clinical trial study with a 2-year follow-up, approved by Institutional Review Board of the Bauru School of Dentistry (CAAE: 24012913.0.1001.5417). The study protocol was registered over the Brazilian Registry of Clinical Trials – REBEC website (#RBR-2jmbvt) and written in accordance with the CONSORT (Consolidated Standards of Reporting Trials) and the SPIRIT (Standard Protocol Items: Recommendations for Interventional Trials) guidelines.

### Sample Size and Study Population

The sample size was calculated through the formula for comparing proportions, considering a power of 80% and a significance level of 5%. According to the literature, the failure proportions were 18% for ART/HVGIC multiple-surface restorations with Ketac Molar and 3% for multiple-surface conventional restorations with resin composite. An additional 20% increase was considered to compensate dropouts, resulting in 77 restorations for each treatment.^18^

The participants of this parallel trial were selected from 17 public primary schools in the countryside of the state of São Paulo, Brazil. The inclusion criteria were as follows: children and adolescents with no medical history; individuals that cooperated for dental examination; one class II cavity in permanent teeth without active periodontal or pulpal diseases or toothache; presence of occluding tooth; good oral hygiene. The included participants were examined using the Caries Assessment Spectrum and Treatment (CAST) instrument,^19^ from which a mean DMFT-score was retrieved.

The exclusion criteria comprised the following: participants presenting mobile teeth, having paranormal occlusion, more than two multiple-surface cavities in permanent teeth and wearing orthodontic appliances.

The size of the cavity was classified as small, medium or large.^18^ Only children or adolescents whose parents or the participant signed the Informed Consent form were included in the study. Stratified randomization was performed by subdividing class II cavities into two homogeneous groups. Cavity size and caries experience (DMFT) were the stratification variables, in that order.

For stratified randomization, due to the difficulty in obtaining the calculated number of class II cavities, the tooth was considered a sample unit. The participants were initially screened and in an Excel spreadsheet the screened teeth were listed with the indication of the cavity size and the patient’s caries index. Teeth were initially ordered in this Excel spreadsheet by the caries index and divided into two conglomerates, the first with the lower DMFT values and the second with the higher values. After this division, in each of these clusters, the teeth were ordered by the size of the cavity and each half was divided into two parts, totaling four clusters namely: low DMFT-scores and small cavity size, low DMFT-scores and large cavity size; high DMFT-scores and small cavity size; and high DMFT-scores and large cavity size.

After this division into four groups, teeth were allocated to groups using the “random” function on Excel to ensure impartiality in the process of randomization and allocation. After this randomization, statistical tests were carried out to ensure that the factors used in the randomization were equally divided among the experimental groups. A T test for independent samples was used for the comparison between the caries index of the two experimental groups and a Mann-Whitney U test to compare the cavity sizes of the two groups (5%).

**Figure 1:**
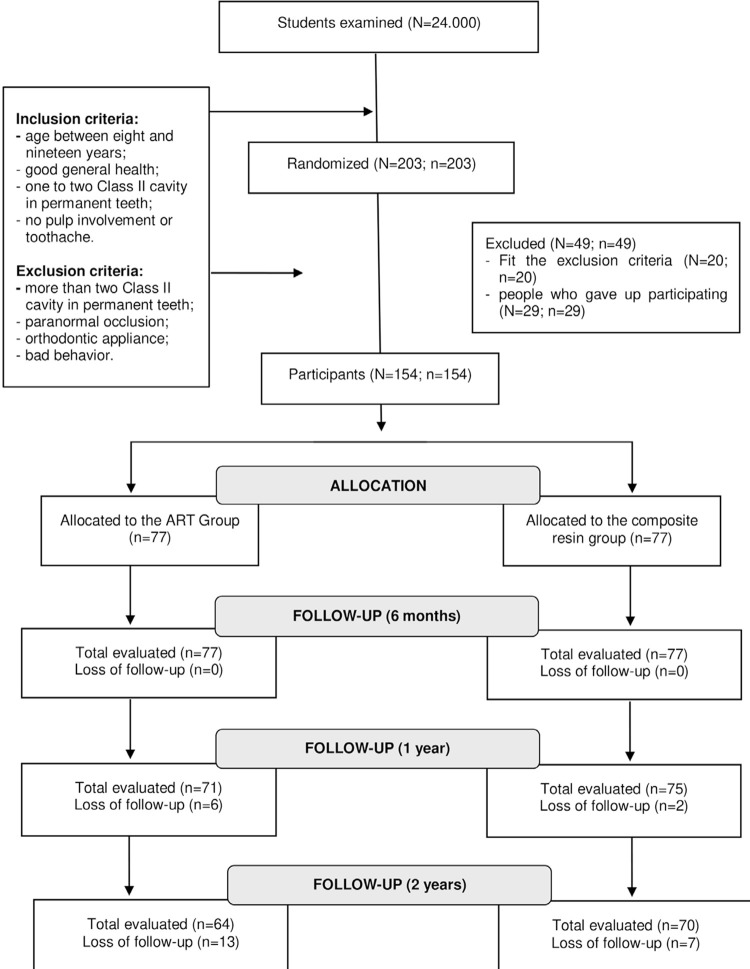
CONSORT flowchart for clinical trials

Figure 1 shows the flow diagram of patient randomization indicated by CONSORT (Consolidated Standards of Reporting Trials).

### Training sessions

Prior to the beginning of the study, a single operator (RMS) was trained in the ART approach, including the creation of additional retentive grooves, and for the resin composite approach. Two experienced dentists in ART and Restorative Dentistry (MFLN and JEF) supervised the training sessions, which involved theoretical, laboratory and clinical exercises. The training was performed in the Clinical Research Center of the Bauru School of Dentistry, Brazil, where the examiners (SRVM and RSB) and assistants were trained for CAST and data recording. One month before each evaluation, the evaluators were trained in using the evaluation criteria by means of theoretical and clinical training.

Inter- and intra-agreements (Kappa coefficient) were performed for caries diagnosis, cavity size classification, and for the evaluation of restorations at 6 months, 1 and 2 years of follow-up, in 10% of the included subjects.

The intra-agreement values for the evaluator 1 for the classifications of caries and size of cavities were 1 and 0.86, respectively. The values for the evaluator 2 were 0.84 and 0.84, respectively. The intra-examiner agreement was 0.85.

For the evaluation of restorations, intra-examiner agreement for evaluator 1 was 0.8 for 6 months and 1 year, and 0.84 for the 2 years follow-up. The values for the evaluator 2 was 0.84 at 6 months, and 0.8 at 1 and 2 years of follow-up. The inter-examiner agreement values were 0.82 for 6 months and 2 years, and 0.80 at the 1-year follow-up.

### Examination

The clinical examinations were performed under adequate lighting. Patients were laid on a table. The examiners seated at a 12-o’clock position and recorders sat at a 9 o’clock position. The dental instruments used were mouth mirrors, wooden spatulas, and the CPI (Community Periodontal Index) probe.

### Restoration Placement

As different techniques and materials were tested, it was not possible to have blindness in this study since the operator, the evaluator and the patient would know which material was being inserted or evaluated. The operator restored by randomization as previously defined and knew the restorative techniques that were being performed; the evaluator was also able to easily identify the two types of restoration and the patient was informed about the materials and techniques that could be used: one with the use of anesthesia and rotating instruments and the other with manual instruments.

The filling materials were used according to the manufacturer’s instructions. ART/HVGIC protocol was described in detail in a previous study.^18^ In summary, hand excavators were used to remove soft dentin and retentive grooves were made in the gingival-occlusal direction in the buccal and lingual walls of the proximal boxes, approximately 0.5 mm from the dentin-enamel junction. The tooth was isolated with cotton rolls. The cavity was cleaned with cotton wool pellets, and conditioned for 15 s with 20% polyacrylic acid. After the placement of a matrix band and wooden wedge, Equia Fil capsules (GC Corporation, Tokyo, Japan) were used to fill the cavity extending slightly over the marginal ridge. Then, the restoration material was held for 40 seconds under pressure. That step resulted in a sealed restoration on the occlusal surface. After 2.5 minutes, hand instruments were used to remove material flashes. The matrix was removed with buccal-lingual and occlusal movements, 5 minutes after the start of GIC mixing. A carbon paper was used to check the occlusion and a dental floss to check the presence of a contact point with the neighboring tooth. Any debris and humidity were removed from the restoration surface and Equia Coat (GC Corporation, Tokyo, Japan) was applied and light cured for 20 s. A schematic drawing of the restoration and its border locations was performed on the clinical form of patients. Patients were recommended not to eat solid food for 1 hour.

Conventional restorations/resin composite protocol: The tooth to be restored was anesthetized and the operative field was isolated with rubber dam. The cavities were prepared using minimal invasive dentistry with # 245 or # 330 carbide burs at high speed. Carious dentin was removed with # 1, 2, or 3 round burs (KG Sorensen, Cotia, Brazil). A gingival marginal trimmer was used to finish the enamel margin in the proximal box. In the case of deep caries, calcium hydroxide cement was applied, followed by the application of a resin-modified glass-ionomer cement (Vitrebond - 3M, Saint Paul, USA). The enamel was etched with 35% phosphoric acid (FGM, Joinvile, Brazil) for 15 s, washed with air/water spray for 20 s and dried with absorbent paper. Afterwards, Scotchbond Universal Adhesive (In Brazil: Single Bond Universal) (3M, Saint Paul, USA) was actively applied for 20 s with a microbrush, air gently sprayed for 5 s and lightcured for 10 s. For restoring the proximal contact and the marginal ridge, a metallic matrix system and Palodent clamp (TDV, Pomarode, Brazil) were used with wood wedge. Oblique increments (up to 2 mm in thickness) of Filtek Z350 XT resin composite (3M ESPE, Saint Paul, USA) were inserted in the proximal box (es), followed by the occlusal box. Each increment was lightcured for 40 seconds with a LED device (Elipar Free Light 2 LED light 3M ESPE, Saint Paul, USA). Excess removal and occlusal adjustment were performed with 12-blade FG 7803F multilayer drills (KG Sorensen, Cotia, Brazil) and T & F 7802 (Jet Carbide Burs, Kyoto, Japan). Polishing was carried out with a 12 and 30-blade multilayer drills (FF9904 from Jet Carbide Burs) and felt discs with the Poligloss paste (TDV, Pomerode, Santa Catarina, Brazil).

### Evaluation

Photographs were taken before and immediately after treatment and at the 6 months, 1 and 2 years, for registration. The restorations were evaluated by two experienced dentists (SRVM and RSB) according to ART^19^ and modified USPHS criteria^20^. For this, they used mouth mirrors, wooden spatulas and the CPI probe.

At each evaluation point, participants received new brushing kits and were guided in oral hygiene. Furthermore, supplementary treatments were offered to participants.

### Statistical Analysis

The chi-square test with linear trend was applied to analyze the distribution of scores according to the ART and the modified USPHS criteria, as well as the percentage of success and failure for ART and resin composite. In addition, Kaplan-Meier test was used to evaluate the survival percentages of the restorations and the difference between survival curves was determined with the Log-Rank test.

The level of significance was set at p<0.05. Statistical analysis was performed with SPSS version 23.0 (Statistical Package for Social Sciences, IBM Inc., USA).

## Results

### In this study, the overall recalls at 2 years was 87%.

ART restorations presented success rates of 98.7% (6 months), 95.8% (1 year) and 90.3% (2 years), and the success rates for conventional restorations were 100% (6-month), 98.7% (1 year) and 91.5% (2 years), according to ART criteria. Significant difference was observed between the restorative approaches at 6 months (*p*=0.033) and 1 year (*p*=0.033) but not at 2 years (p=0.064) (Table 1).

**Table 1:** Distribution of scores according to the ART criterion for ART restorations with HVGIC and conventional restorations with resin composite

Scores*	6 months			1 year			2 years		
	**ART restorations n(%)**	**Conventional restorations n (%)**	p**	**ART restorations n(%)**	**Conventional restorations n (%)**	p**	**ART restorations n(%)**	**Conventional restorations n (%)**	p**
(1) Restoration present and correct	65 (84.4)	74 (96.1)	0.033	56 (78.9)	73 (97.4)	0.003	43 (67.2)	59 (84.3)	0.064
(2) Small marginal defect and/or wear with less than 0.5 mm; without need of repair	11 (14.3)	3 (3.9)		12 (16.9)	1 (1.3)		13 (20.3)	5 (7.2)	
									
(3) Marginal Defect exceeding 0.5 mm. Need of repair	-	-		1 (1.4)	0 (0.0)		-	-	
(4) Wear exceeding 0.5 mm. Need of repair	-	-		1 (1.4)	0 (0.0)		-	-	
(6) Restore and/or fracture tooth. Need of repair	1 (1.3)	0 (0.0)		1 (1.4)	1 (1.3)		2 (3.1)	4 (5.7)	
(7) Restoration has completely disappeared. Treatment is needed	-	-		-	-		1 (1.6)	1 (1.4)	
(9) Tooth has been extracted	-	-		-	-		2 (3.1)	0 (0.0)	
(10) Sensitivity or pulpal involvement	-	-		-	-		3 (4.7)	1 (1.4)	

*1 and 2 = success; 3, 4, 6, 7 and 10 = failure; 9 = excluded. **Chi-square test with linear trend.

According to the modified USPHS criterion, the success rates for ART restorations were 98.7% (6 months), 95.8% (1 year) and 92.0% (2 years), and for conventional restoration were 100% (6 months), 98.7% (1 year) and 91.5% (2 years). There was a significant difference between ART with HVGIC and conventional restoration with resin composite at the 6 months’ evaluation (*p*=0.001) but not after 1 year (*p*=0.310) and 2 years (*p*=0.830) (Table 2).

**Table 2:** Distribution of scores according to the modified USPHS criterion for ART restorations with HVGIC and conventional restorations with resin composite

Clinical parameters	Rating	6 months			1 year			2 years		
		**ART restorations n(%)**	**Conventional restorations n (%)**	**p**	**ART restorations n(%)**	**Conventional restorations n (%)**	**p**	**ART restorations n(%)**	**Conventional restorations n (%)**	**p**
Color	Alpha	33 (43.4)	55 (71.4)	< 0.001	32 (45.0)	38 (51.4)	0.609	31 (50.0)	35 (53.0)	0.368
	Bravo	43 (56.6)	22 (28.6)		39 (55.0)	36 (48.6)		31 (50.0)	31 (47.0)	
Marginal discoloration	Alpha	75 (98.7)	74 (96.1)	0.315	65 (91.5)	70 (94.6)	0.785	56 (90.3)	62 (94.0)	0.294
	Bravo	1 (1.3)	3 (3.9)		6 (8.5)	4 (5.4)		6 (9.7)	4 (6.0)	
Relapse of caries	Alpha	76 (100.0)	77 (100.0)	-	69 (97.2)	74 (100.0)	0.235	59 (95.2)	64 (97.0)	0.221
	Charlie	0 (0.0)	0 (0.0)		2 (2.8)	0 (0.0)		3 (4.8)	2 (3.0)	
Anatomical shape	Alpha	43 (56.6)	70 (90.9)	<0.001*	45 (63.4)	66 (89.2)	< 0.001 *	32 (51.6)	54 (81.8)	0.001*
	Bravo	33 (43.4)	7 (9.1)		23 (32.4)	8 (10.8)		29 (46,.8)	10 (15.2)	
	Charlie	0 (0.0)	0 (0.0)		3 (4.2)	0 (0.0)		1 (1.6)	2 (3.0)	
Marginal integrity	Alpha	70 (90.9)	72 (93.5)	0.471*	56 (78.9)	69 (92.0)	0.072 *	50 (80.6)	56 (80.0)	0.361*
	Bravo	6 (7.8)	5 (6.5)		12 (16.9)	5 (6.7)		10 (16.1)	8 (11.4)	
	Charlie	0 (0.0)	0 (0.0)		2 (2.8)	0 (0.0)		0 (0.0)	2 (2.9)	
	Delta	1 (1.3)	0 (0.0)		1 (1.4)	1 (1.3)		2 (3.3)	4 (5.7)	
Surface texture	Alpha	42 (55.3)	63 (81.8)	<0.001	32 (45.1)	56 (75.7)	< 0.001	32 (51.6)	48 (72.7)	0.022
	Bravo	34 (44.7)	14 (18.2)		39 (54.9)	18 (24.3)		30 (48.4)	18 (27.3)	
Restoration quality**	Ideal	25 (32.5)	47 (61.0)	0.001*	27 (38.0)	36 (48.0)	0.310*	21 (33.9)	27 (38.6)	0.830*
	Satisfactory	51 (66.2)	30 (39.0)		41 (57.8)	38 (50.7)		36 (58.1)	37 (52.9)	
	Unsatisfactory	1 (1.3)	0 (0.0)		3 (4.2)	1 (1.3)		5 (8.0)	6 (8.5)	

*Chi-square test with linear trend. **The ideal and satisfactory scores = success; unsatisfactory = fail.

At the end of 2 years, two ART restorations received the score 9 according to ART criterion, being excluded from the analysis. Those restorations were also excluded from the evaluation with the modified USPHS criterion. Those restorations with code 9 (ART criterion) can be identified in Table 2. It is important to note that in this same evaluation period, another ART restoration classified with the score 6 according to the ART criterion was considered satisfactory as stated in the modified USPHS criterion (Tables 1 and 2).

Regardless of the evaluation criteria used, the success rates for conventional restoration with resin composite were the same. However, considering the success rates for ART restorations with HVGIC after 2 years, they were 90.3% when using the ART criterion and 92.0% when using the modified USPHS criterion, with no difference between them (*p*<0.466).

During the 6-month recall, ART differed from the conventional approach only within the clinically acceptable scores, on color, anatomical form, and surface texture (p<0.001). After 1- and 2-year recalls, differences were detected for anatomical form and surface texture (p<0.022) (Table 2).

**Figure 2:**
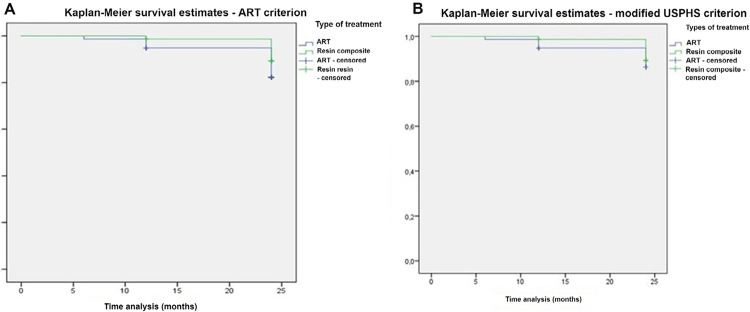
Graphs for survival analysis of ART restorations with HVGIC and conventional restorations with resin composite, at the 2-year follow-up. Graphs represent data assessed with the ART or the modified USPHS criteria

The survival percentages for ART restorations assessed by the ART criteria were 98.7% (6 months), 94.8% (1 year) and 83.7% (2 years), and for the conventional restorations the percentages were 100 % (6 months), 98.7% (1 year) and 90.7% (2 years). There was no difference in the survival curves of ART restorations with HVGIC and conventional restorations with resin composite after 2 years (p = 0.181) (Figure 2).

Considering modified USPHS criterion, survival percentages of ART restorations were 98.7% (6 months), 94.8% (1 year) and 87.8% (2 years), and for the conventional restoration 100% (6 months), 98.7% (1 year) and 90.7% (2 years). There was no difference in the survival curves of ART restorations with HVGIC and conventional restorations with resin composite after 2 years (p = 0.552) (Figure 2).

**Figure 3:**
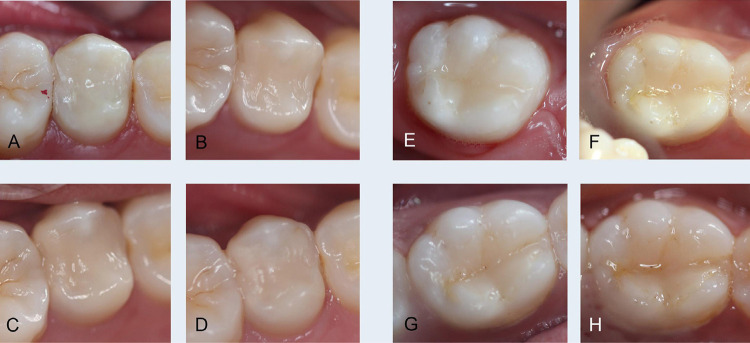
Class II (DO) ART restoration with HVGIC in the upper left second premolar: A) Baseline; B) 6 months; C) 1 year and D) 2 years and Conventional Class II (MO) restoration with resin composite in mandibular right first molar: E) Baseline; F) 6 months; G) 1 year and H) 2 years

Figure 3 illustrates the two types of restorative treatment in this study.

## Discussion

Both HVGIC and resin composite presented high success rates after 2 years (>90%). Although clinical success was similar within the assessed period of time, one might consider performing ART restorations since it has some advantages, among them the use of inexpensive hand instruments, only infected tooth tissue being removed, the employed material presenting chemical adhesion to the tooth substrates, and fluoride release.^19^

In the present study, regarding ART restorations, the average annual failure rate was 6.3%, presenting a longevity of restorations higher than a metanalysis that reported 78.2%^21^. The difference can in part be attributed to the material used, which was an encapsulated GIC, presenting improved mechanical properties compared with hand-mixed GICs commonly used in ART.^7^ The present material also contains improved liquid and powder and the restoration surface is coated with nanofilled resin. The encapsulated GIC removes the negative effects of proportioning the powder/liquid ratio and diminishes the number of porous produced by hand-mixing. In addition to encapsulation, Equia Fil combines the main advantages of HVGIC, with a nanofilled, light cured coat, which protects the material in the initial setting phase and occludes surface cracks and porosity, increasing its wear resistance and toughness.^12,14,22,23^

Other very important highlighted aspects for the present study are the time lapsed from the beginning of mixing and the initial removal of material excess (2.5 minutes) and matrix removal (5 minutes). Those are important to allow the initial material setting and hardening.^24^ On the other hand, longer waiting periods are not desired due to difficult excess removal with possible occlusal interferences being left, a fact that would lead to early restoration fracture. The operator was aware and took into consideration bonding stability of HVGICs to dentin is not so strong in the early periods.^25^ Moreover, a step that may also increase the longevity of multi-surface HVGIC restorations was the creation of retention grooves in proximal boxes, close to the amelodentinal junction, as noted by Barata, et al.^5^ (2008) and Fernandes, et al.^6^ (2019).

Regarding conventional restoration with resin composite, the average annual failure rate was 4.3%, being within the reported mean, which varies from 2 to 10% depending on the adhesive strategy used.^26-28^

Generally, ART restorations are evaluated by ART criteria in most studies whilst the longevity of restorations are assessed by USPHS criterion.^15,29^ It has been suggested that the ART criteria are more stringent than the USPHS criteria, since the marginal defect or wear exceeding 0.5 mm is considered to be a failure in the ART criteria and not for the USPHS criteria, which will consider failure only if dentin is exposed.^30,31^ Moreover, the ART criteria of dental fracture considers failure even if the restoration remains intact, opposing the USPHS criterion that considers this scenario as success. Our results showed that regardless of the evaluation criteria used for restorations, the success rates were identical or similar, and there was no statistical difference between them according to the findings in the literature.^29-31^ This was likely because the only two restorations with scores 3 and 4 (ART criteria) were also considered failures according to the USPHS criteria.

This study considered the use of the modified USPHS criteria adequate and comparable to the ART criteria. These criteria are relevant since they can assess marginal discoloration, color and surface texture, which are not measured by the ART criteria.^32^

At the 6-month evaluation, differences were detected within the clinical acceptable parameters for both treatments (color, anatomical shape and surface texture). After 1 and 2 years, differences in anatomical shape and surface texture were detected between the restorative approaches (Table 2). Differences in color were lost after the 6-month recall. Although there were differences between the restorations and their anatomical forms and superficial textures, during the 2 years of follow up, the quality of the restorations was not compromised. Since the anatomy of ART restorations is achieved by digital pressure, their anatomical shape would be a disadvantage over conventional restorations with resin composite. Besides the resin composite being nanoparticulated, which ensures a high surface smoothness, the polishing of restorations performed also collaborate to a smoother surface texture, possibly explaining the differences found in surface texture between treatments.

The main reasons for failure in this study according to the ART criterion were: fracture of the restoration and/or tooth (9 restorations), painful symptomatology (5 restorations), one restoration failed due to excessive wear and one restoration failed due to a marginal defect of more than 0.5 mm. Only after 2 years one restoration fail due to secondary caries. The other fractures in the study probably occurred due to different intrinsic reasons of restorative materials, the technique employed or patient habits.^18,33^

The results of the present study showed that the preventive effect of caries was similar for both materials. After 2 years, abscess and/or fistula were present in three ART restorations and two conventional restorations, suggesting a high level of efficacy after 2 years, regardless of the high caries experience of the participants (DMFT=4.72). It is worth noting that for those restorations, the protection of the dentin-pulp complex with calcium hydroxide solution and cement had been applied for the ART restorations, while calcium hydroxide solution and cement and GIC base were applied when necessary for conventional restorations with composite resin. Six other teeth with deep carious lesions such as these responded favorably to the protection of the dentin-pulp complex, maintaining pulp vitality and restorative success after 24 months.

One study reported^34^ that after a three-year follow-up, the annual failure rates for resin composites and resin-modified GICs in deep cavities were 14.6% and 26.7%, respectively. Now, regarding the management of carious lesions, the annual failure rates for the restorations were 17.3% when the selective removal was performed and 13.1% when the total removal of the carious lesion was performed. Thus, in the present study, failure rates, especially for painful symptomatology due to pulp involvement, were considered low.

The present results do not follow the main reasons for the substitution of conventional restorations with resin composite observed in the literature, which are the development of secondary caries related to the adhesive interface and fracture of the restoration that is related to the mechanical properties of the material, as well as to the quantity and quality of the remaining dental structure.^35,36^

Poor oral hygiene along with high rates of DMFT negatively impact the success of restorations in general.^37^ Most publications do not report the oral health status of subjects included in the studies nor did they report whether a dental health program was implemented along with clinical treatment.^37^ This may also have been one of the reasons for the observed restorative success of this study, because in addition to explaining how to clean their mouth and reinforcing the importance of preventive measures in each follow-up, the participants received a new kit for oral hygiene at each evaluation point.

By comparing the success rates of treatments (data raw) and the survival analysis values (from a probability curve), we observed that the survival analysis generally provided a lower restorative success for both approaches tested in the study. At this follow-up period, although the survival analysis presents lower values, they are close to the raw percentages detected. The authors are not aware if this trend would be more perceptible in future recalls. To our understanding, the survival analysis underestimates the real effectiveness of restorations in non-inferiority studies.^38-40^

The results of the present study showed that the null hypothesis could not rejected, and there is no difference after 2 years on the effectiveness of these ART restorations with HVGIC compared to conventional restorations with resin composite.
